# Insights into Biodegradation Related Metabolism in an Abnormally Low Dissolved Inorganic Carbon (DIC) Petroleum-Contaminated Aquifer by Metagenomics Analysis

**DOI:** 10.3390/microorganisms7100412

**Published:** 2019-10-01

**Authors:** Pingping Cai, Zhuo Ning, Ningning Zhang, Min Zhang, Caijuan Guo, Manlan Niu, Jiansheng Shi

**Affiliations:** 1 Institute of Hydrogeology and Environmental Geology, Chinese Academy of Geological Sciences, Shijiazhuang 050061, China; cppyjy@163.com (P.C.); ningzhuozhuo@163.com (Z.N.); 2105170079@cugb.edu.cn (N.Z.); caizidongdong@163.com (C.G.); tiger7886@263.net (J.S.); 2 School of Resources and Environmental Engineering, HeFei University of Technology, Hefei 230009, China; hfnml@163.com; 3 Key Laboratory of Groundwater Remediation of Hebei Province, Zhengding 050083, China; 4 School of Water Resources and Environment, China University of Geosciences (Beijing), Beijing 100083, China

**Keywords:** microbial metabolism, petroleum-contaminated aquifer, metagenomics analysis

## Abstract

In petroleum-contaminated aquifers, biodegradation is always associated with various types of microbial metabolism. It can be classified as autotrophic (such as methanogenic and other carbon fixation) and heterotrophic (such as nitrate/sulfate reduction and hydrocarbon consumption) metabolism. For each metabolic type, there are several key genes encoding the reaction enzymes, which can be identified by metagenomics analysis. Based on this principle, in an abnormally low dissolved inorganic carbon (DIC) petroleum-contaminated aquifer in North China, nine groundwater samples were collected along the groundwater flow, and metagenomics analysis was used to discover biodegradation related metabolism by key genes. The major new finding is that autotrophic metabolism was revealed, and, more usefully, we attempt to explain the reasons for abnormally low DIC. The results show that the methanogenesis gene, *Mcr*, was undetected but more carbon fixation genes than nitrate reduction and sulfate genes were found. This suggests that there may be a considerable number of autotrophic microorganisms that cause the phenomenon of low concentration of dissolved inorganic carbon in contaminated areas. The metagenomics data also revealed that most heterotrophic, sulfate, and nitrate reduction genes in the aquifer were assimilatory sulfate and dissimilatory nitrate reduction genes. Although there was limited dissolved oxygen, aerobic degrading genes *AlkB* and *Cdo* were more abundant than anaerobic degrading genes *AssA* and *BssA*. The metagenomics information can enrich our microorganic knowledge about petroleum-contaminated aquifers and provide basic data for further bioremediation.

## 1. Introduction

The biodegradation of petroleum plays a vital role in transforming hydrocarbons into harmless matter in the natural environment [1]. The understanding of microorganism metabolism related to biodegradation in petroleum hydrocarbon contaminated (PHC) aquifers can help us to evaluate the potential ability of biodegradation, which is the theoretical basis of bioremediation [2].

It is generally believed that the biodegradation of petroleum hydrocarbons consists of oxidoreductase biochemical reactions caused by microorganisms in aquifers. In the reactions, petroleum hydrocarbons are considered to be electron donors, while dissolved oxygen (O_2_) and inorganic oxide, such as nitrate (NO_3_^−^), manganese (IV) (Mn^4+^), ferric ion (Fe^3+^), sulfate (SO_4_^2−^), and CO_2_, are considered to be electron acceptors [3]. In these biochemical reactions, metabolism related to organic carbon consumption, such as nitrogen and sulfur metabolism, are involved. Along with biodegradation, methane generation, and other types of carbon fixation metabolism may also exist in petroleum-contaminated aquifers [4,5].

To reveal such metabolism in PHC aquifers, many studies have been carried out. Most of these studies focused on the relationship between microbial community diversity and environmental factors, and it is generally acknowledged that hydrocarbon biodegradation microorganisms vary depending on sampling depth, contaminant concentration, redox conditions, groundwater flow field conditions, and so on [6,7,8]. Community diversity studies give us information about microbial species, but do not provide direct evidence of a community’s functional capabilities, which is crucial for understanding the roles that the microorganisms play [9]. Around the issue, many functional genes, including hydrocarbon oxygenase genes (e.g., *AlkB*, *AssA*, and *BssA*), methanogenic genes (e.g., *Mrc*), and carbon fixation genes (e.g., *RubisCO*), as well as key genes of the nitrogen/sulfur cycle (e.g., *NosZ,* and *Dsr*) were explored [4,10,11,12,13,14,15]. Typically, these studies either focused on quantifying the limited genes by quantitative PCR or taking some related genes as a whole but in qualitative ways using GeoChip. A systematic quantification of the dynamics of microbial metabolism genes associated with petroleum-contaminated environments is still lacking. In recent years, Reid et al. [16] quantitatively revealed various functional variations of microbial ecosystems in freshwater hydrocarbon-rich river sediment using metatranscriptomics. This marked the beginning of new insights into biodegradation related metabolism. Especially for some unexplained mechanisms, it is possible to produce new direct microbial metabolism evidence.

Fittingly, contrary to the general law that dissolved inorganic carbon (DIC) is usually affected by organic carbon consumption metabolism producing DIC, an abnormal phenomenon of low DIC, possibly caused by carbon fixation, was discovered in a PHC aquifer in North China. Biodegradation related metabolism might well explain that abnormal phenomenon. To obtain sufficient and reliable evidence, nine monitoring wells along the groundwater flow were sampled. A metagenomics approach combined with next-generation sequencing was carried out to obtain a broad-spectrum profile of microbial information in collected samples. Functional processes related to energy metabolism, including carbon fixation pathways in prokaryotes; nitrogen, sulfur, and methane metabolism; and hydrocarbon degradation, were analyzed based on the metagenomics data. The results could give us a new knowledge about petroleum-contaminated aquifers and be helpful for the management of these contaminated sites.

## 2. Materials and Methods

### 2.1. Site Description

**Figure 1:**
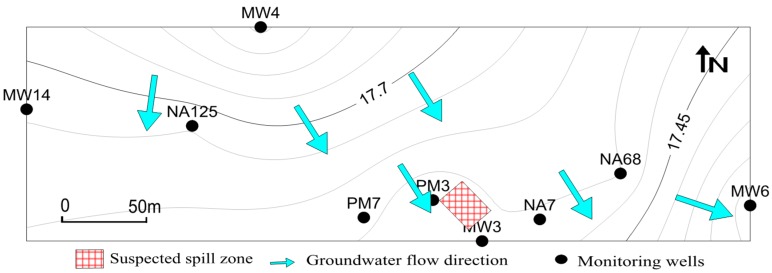
Map showing suspected spill zone, monitoring wells, and groundwater flow.

The sampling site is located at a former petrochemical plant in the piedmont plain of North China. The site was contaminated decades ago by petroleum leaked from storage tanks. The contaminants on the surface have been removed, but the groundwater at the site is still contaminated by petroleum. The groundwater flow is from northwest to southeast/east. Along the groundwater flow, monitoring wells were designed in and around the leakage zone to monitor the information of contaminated groundwater (Figure 1). There was no groundwater around the site that was free from contamination according to our previous study. Monitoring well MW 14 is far away (about 200 m) from and upstream of the spill zone and had the least contaminants but greater concentrations of terminal electron acceptors (DO, NO_3_^−^, and SO_4_^2^^−^). Therefore, MW14 can be considered as the background well. Along the groundwater flow, MW4 and NA125 were designed as the upstream wells, and PM3 and MW3 wells were designed as the contaminated source wells, while NA68 and MW6 were designed as the downstream wells. PM7 and NA7 were designed as transitional wells to monitor the information of the zone adjacent to the contaminated source zone.

The aquifer at the site is mainly composed of sand gravel layers. The thickness of the aquifer is about 3.3 m. Its lower layer is silty clay, which can be considered as the aquiclude. Its upper layer is also silty clay at most of the site, while in the downstream area (around NA68 and MW6) there is no upper aquiclude. The groundwater depth is about 21.7 m to 24.0 m and the water table is higher than the upper confining bed. Therefore, the groundwater is confined in the main area and unconfined in the downstream area. The groundwater flow is from northwest to southeast.

### 2.2. Sample Collection and Chemical Analysis

Samples were collected from the monitoring wells using bailers in August 2018. Information about the water temperature (T), pH, electrical conductivity (EC), dissolved oxygen (DO), and oxidation–reduction potential (ORP) in the collected groundwater was determined using a Portable Parallel Analyzer (SL1000, Hach, Loveland, CO, USA) and recorded before collecting the laboratory testing samples. The groundwater samples were considered to be representative when the variations of T, pH, EC, DO, and ORP in three successive samples were within ±1 °C, ±0.2, ±3%, ±10%, or ±0.2 mg/L, and ±20 mV, respectively. When sampling, 1 L of groundwater was collected in two 500 mL plastic buckets for inorganic analysis and 160 mL was collected in four 40 mL amber glass bottles with Teflon airtight caps for organic and CO_2_ analysis. The samples were then stored on ice in an incubator before being transported to the laboratory. For the metagenomics analysis, 5 L of groundwater was collected in a sterilized 5 L plastic bucket and stored on ice in an incubator. The water samples were transported to the laboratory, and DNA was collected in five poly tetra fluoroethylene filter membranes with a pore size of 0.22 μm by air pump filtration in one day. The filter membranes were stored at −80 °C until DNA extraction.

Concentrations of the monocyclic aromatics benzene, toluene, ethylbenzene, m-xylene, p-xylene, and o-xylene (BTEX), as well as volatile organic compounds (VOCs), were determined according to the US Environmental Protection Agency (EPA) Methods 8260 and 8015d [17,18]. Concentrations of other parameters of the groundwater, namely chemical oxygen demand (COD), pH, Ca^2+^, Mg^2+^, Na^+^, K^+^, NO_3_^−^, SO_4_^2−^, HCO_3_^−^, Cl^−^, Fe^2+^, Mn^2+^, and CO_2_, were measured following standard methods [19].

### 2.3. DNA Isolation, Sequencing, and Library Construction

Total genomic DNA was extracted from nine collected samples using the E.Z.N.A.^®^ DNA Kit (Omega Bio-tek, Norcross, GA, USA) according to the manufacturer’s instructions. Concentration and purity of extracted DNA were determined with TBS-380 and NanoDrop2000, respectively. DNA extract quality was checked on 1% agarose gel.

DNA extract was fragmented to an average size of about 300 bp using Covaris M220 (Gene Company Limited, Hong Kong, China) for paired-end library construction. A paired-end library was constructed using a TruSeq^TM^ DNA Sample Prep Kit (Illumina, San Diego, CA, USA). Adapters containing the full complement of sequencing primer hybridization sites were ligated to the blunt ends of fragments. Paired-end sequencing was performed on an Illumina HiSeq4000 platform (Illumina Inc., San Diego, CA, USA) at Majorbio Bio-Pharm Technology Co., Ltd. (Shanghai, China) using a HiSeq 4000 PE Cluster Kit and HiSeq 4000 SBS Kit according to the manufacturer’s instructions (www.illumina.com).

### 2.4. Bioinformatics Analysis

(1) Sequence quality control and genome assembly:

Adapter sequences were stripped from the 3’ and 5’ ends of paired-end Illumina reads using SeqPrep (https://github.com/jstjohn/SeqPrep). Low-quality reads (length <50 bp or quality value <20 or having N bases) were removed by Sickle (https://github.com/najoshi/sickle). Metagenomics data were assembled using MEGAHIT (https://github.com/voutcn/megahit), which makes use of succinct de Bruijn graphs. Contigs with a length of 300 bp or more were selected for the final assembling result, and then the contigs were used for further gene prediction and annotation.

(2) Gene prediction, taxonomy, and functional annotation:

Open reading frames (ORFs) from each assembled contig were predicted using MetaGene (http://metagene.cb.k.u-tokyo.ac.jp/). The predicted ORFs with a length of 100 bp or more were retrieved and translated into amino acid sequences using the National Center for Biotechnology Information (NCBI) translation table.

All predicted genes with a 95% sequence identity (90% coverage) were clustered using CD-HIT (http://www.bioinformatics.org/cd-hit/), and the longest sequences from each cluster were selected as representative sequences to construct a non-redundant gene catalog. Reads after quality control were mapped to the representative sequences with 95% identity using SOAPaligner (http://soap.genomics.org.cn/), and gene abundance in each sample was evaluated.

Representative sequences of the non-redundant gene catalog were aligned to the NCBI NR database with an e-value cutoff of 10^−5^ using BLASTP (version 2.2.28+, http://blast.ncbi.nlm.nih.gov/Blast.cgi) for taxonomic annotation.

Functional read annotations for presented figures and tables were assigned using the Enzyme database of the Kyoto Encyclopedia of Genes and Genomes (KEGG). Gene expression pathways were explored/visualized using the KEGG mapper [16]. Parts per million (ppm), or reads annotated as enzymes in one million sequencing reads, was used to represent the relative abundance of genes encoding enzymes in samples [20].

## 3. Results and Discussion

### 3.1. Hydrochemical Characteristics of Groundwater

Contaminant concentrations, electron acceptors, metabolic byproducts, and other hydrochemical parameters of the collected samples from August 2018 are listed in Table 1.

All the contamination indices indicated that the aquifer was still contaminated by petroleum hydrocarbons. There was no sample free from contamination in the aquifer. The downstream wells, MW3, NA7, NA68, and MW6, had higher concentrations of contaminants. This indicates that contaminants migrated to the downstream aquifer with groundwater flow.

DO and SO_4_^2−^ concentrations and ORP values had similar distribution: Values were higher upstream and decreased with groundwater flow, while Fe^2+^ and Mn^2+^ concentrations were just the opposite. This indicates that there was biodegradation of hydrocarbons, as the electron acceptors were consumed [3]. NO_3_^−^ should have had similar distribution according to the natural attenuation theory [21], but it did not tally with the facts. There was more NO_3_^−^ upstream and downstream but less in the contaminated source area. The most likely cause is that there was outside nitrogen input around NA68. The evidence is that there is a sewage treatment plant nearby and the ammonium concentration in NA68 is particularly high (300 mg·L^−1^). Even though there was nitrogen input, the concentration decreased compared with the upstream wells. It is suggested that there was also nitrate reduction biodegradation. Our previous study revealed that biodegradation in the aquifer in 2016 was mainly caused by nitrate and sulfate reduction [22]. It was also true in 2018, according to the variations of electron acceptor concentrations listed in Table 1.

Dissolved inorganic carbon (DIC) concentration in wells around the contamination source zone (NA125, PM7, PM3, and MW3) was lower than that in upstream and downstream wells. It is generally acknowledged that heterotrophic microorganisms completely oxidize organic carbon (hydrocarbons) to inorganic carbon (CO_2_), elevating DIC as a result [23]. Most studies have found that there are higher DIC concentrations in organically contaminated groundwater worldwide [24,25,26,27]. PM7, PM3, and MW3 had more hydrocarbons than upstream wells and were expected to have higher DIC concentrations, which seems to conflict with the statement above. The phenomenon of abnormally low DIC concentration in the petroleum-contaminated aquifer attracted our attention when analyzing the hydrochemical parameters of the groundwater collected in August 2016 at the same site. It is speculated that the phenomenon was caused by carbon fixation of autotrophic metabolism through the sequencing of 16S rRNA gene [28]. Other researchers have found that autotrophic microorganisms may exit in PHC groundwater [5,29]. Whether there are carbon fixation microorganisms in the aquifer should be revealed by metagenomics analysis. Although the lack of samples free from contamination in the aquifer make us lose the opportunity to assess the differences of metagenomes between contaminated and non-contaminated areas, the samples collected in different contaminated areas along the groundwater flow had different characteristics of contamination and hydrochemistry as analyzed above. Therefore, there would be some interesting findings about microorganism metabolisms in different groundwater flow-field locations through the analyses of samples we collected.

### 3.2. Sequencing Statistics

The Illumina HiSeq 4000 sequencing resulted in over 50 million sequence counts for all samples. More than 300,000 contigs were selected for gene prediction and annotation. According to the NCBI NR database, genes were annotated at the domain level, as shown in Table 2.

Table 2 shows that more than 97% of the sequences were annotated to bacteria in all samples. This suggests that the organisms in the aquifer were dominated by bacteria.

### 3.3. Metabolism Analysis

#### 3.3.1. Carbon Fixation Metabolism

There are at least six carbon fixation pathways known to exist in autotrophic bacteria and archaea: The Calvin-Benson-Bassham (CBB) cycle, Wood-Ljungdahl (WL) pathway, dicarboxylate-hydroxybutyrate (DH) cycle, 3-hydroxypropionate (HP) cycle, Arnon-Buchanan (AB) cycle, and hydroxypropionate-hydroxybutyrate (HH) cycle [30,31]. The carbon fixation enzymes in these pathways were gained from the map of “carbon fixation pathways in prokaryotes” in the KEGG PATHWAY Database. Since HP and HH share the same carbon fixation enzymes [31], these pathways were analyzed together. The science of DH partly involves carbon fixation enzymes of the AB cycle as well as enzymes of the HH cycle, and there are no enzymes that are unique to the DH pathway combined with the limited microorganisms in the DH pathway, to our knowledge [31]; the DH pathway was not discussed alone in the study.

**Figure 2:**
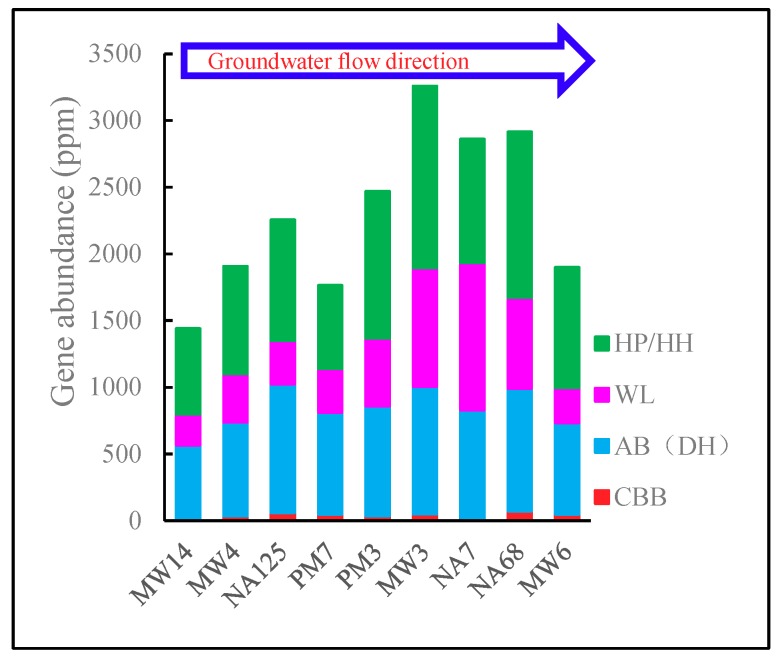
Distribution of carbon fixation genes. HP, 3-hydroxypropionate; HH, hydroxypropionate-hydroxybutyrate; WL, Wood-Ljungdahl; AB, Arnon-Buchanan; DH, dicarboxylate-hydroxybutyrate; CBB, Calvin-Benson-Bassham.

The abundance of genes encoding these enzymes is listed in Supplementary Table S1. The total abundance of genes encoding transformation of CO_2_ to other material in each pathway is shown in Figure 2. The AB (DH), HP/HH, and WL pathway genes were more abundant than the CBB pathway genes. It was an interesting result that the CBB pathway, always considered as the most important carbon fixation pathway and dominant in most ecosystems [12], had the least abundance among all the pathways. The lower abundance of CBB carbon fixation genes indicates that petroleum hydrocarbons may change the aquifer ecosystem and stimulate carbon fixation microorganisms in other pathways.

Another interesting finding is that the carbon fixation genes were more abundant in the contaminated source area, which was attributed to the higher abundance of carbon fixation genes in the WL and HP/HH pathways. The analysis of species structure (Supplementary Figure S1) showed that most of the dominant species involved in carbon fixation, such as *Reyranella, Novosphingobium*, *Microbacterium, Sphingomonas*, *Acidovorax*, and *Bordetella,* potentially have the ability to biodegrade hydrocarbons [32,33,34,35,36,37,38,39,40]. It is also reported that these bacteria can assimilate inorganic carbons and be considered as facultative autotrophic microorganisms [41,42]. It is reported that carbon fixation pathways, e.g., the AB, or HP pathway, may facilitate the assimilation of simple organic substances, e.g., acetate, succinate, or propionate, as these substances are intermediates of these pathways. Thus, carbon mixotrophs using such pathways have a competitive advantage over obligate autotrophs or heterotrophs [31]. Therefore, these facultative autotrophic microorganisms thrive in the aquifer, especially in the contaminated source zone.

Although the mechanism is still unclear and should be studied further, the result explains the phenomenon of low DIC concentrations in the contaminated aquifer. NA125, PM7, and PM3 wells were less contaminated, and they had lower DIC concentration. In particular, the NA125 well had the most abundant carbon fixation genes among upstream wells (MW14, MW4, NA125, and PM7) that had fewer contaminants, and therefore had the lowest DIC concentration (Table 1). MW3, NA7, and NA68, the most contaminated wells, had a higher abundance of carbon fixation genes, but with the DIC generated by heterotrophic metabolism, their DIC concentrations were slightly elevated. MW6 had fewer carbon fixation genes but more contaminants, which resulted in heterotrophic metabolism dominating the area and elevated DIC concentration.

The amount of genes encoding carbon fixation was more than 1500 ppm in all samples. Except for MW6, the abundance of carbon fixation genes was greater than that of genes involved in nitrate and sulfate reduction (Supplementary Figure S2), both of which are the primary biodegradation reactions in petroleum-contaminated groundwater [22,43]. Therefore, there would be a big carbon sink effect in the aquifer. The carbon fixation metabolism should be considered in future studies of petroleum-contaminated aquifers.

#### 3.3.2. Methane Metabolism

**Figure 3:**
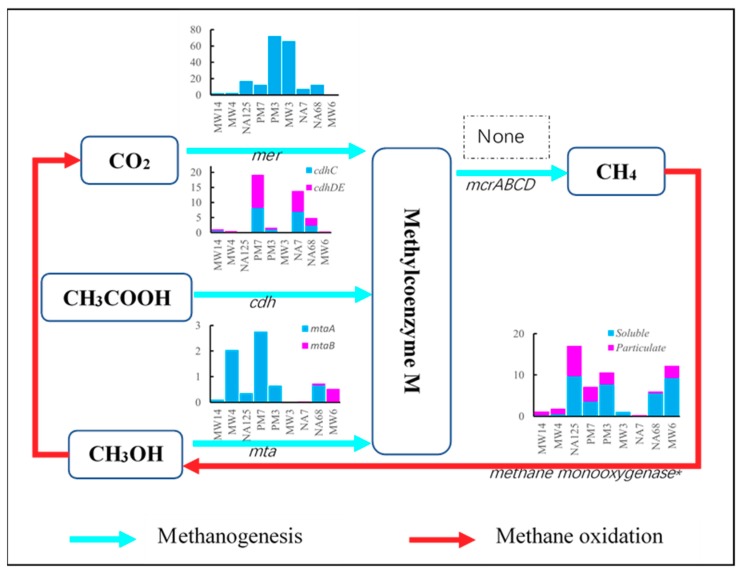
Gene abundance of methane metabolic pathways in the aquifer.

Methane metabolism includes methanogenesis and methane oxidation. Methanogens can obtain energy for growth by converting a limited number of substrates to methane under anaerobic conditions. Three types of methanogenic pathways are known: CO_2_ to methane, methanol to methane, and acetate to methane [44]. According to the KEGG pathway map, all the methanogenic pathways can generate the same intermediate product, methylcoenzyme M, which will be reduced to methane by methyl-coenzyme M reductase (Figure 3). The abundance of *mer*, *cdh*, and *mta* is ~70, ~20, and ~3 parts per million (ppm), respectively. Therefore, the pathway of CO_2_ to methylcoenzyme M is dominant. This process mainly occurs around the contaminated source area (PM3 to MW3). It may be because there was less dissolved oxygen (DO) in the area (Table 1), making it suitable for the reduction of CO_2_ [45]. According to Figure 3, there was no detected *mcr* gene encoding methyl-coenzyme M reductase, which is associated with the final stage of methanogenesis. That may because there was no methanogenic redox environment or there were other adequate preferred electron acceptors for microorganisms (Table 1). Therefore, it is suggested that little methane would be generated from the aquifer and released to the atmosphere.

However, there was some methane monooxygenase gene, a key methane oxidation gene, in collected samples. It is suggested that there was methanogenesis in the aquifer, but the gene was undetectable. MW3 and NA7 had the lowest methane monooxygenase gene abundance because of the limited oxygen, while MW4 and MW14 had lower methane monooxygenase gene abundance because of the limited methane upstream.

#### 3.3.3. Nitrogen Metabolism

**Figure 4:**
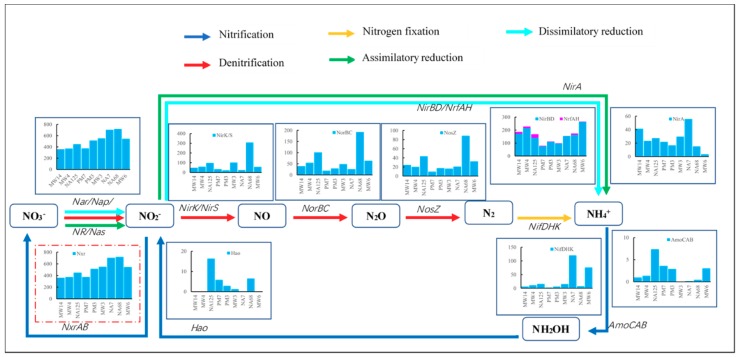
Gene abundance of nitrogen metabolism pathways in the aquifer.

There are three methods of nitrate reduction, according to the KEGG map: Denitrification, dissimilatory reduction, and assimilatory reduction (Figure 4).

In all three nitrate reduction methods, the nitrate should first be transformed into nitrite. The enzyme in this step is nitrate reductase. Genes encoding this enzyme are *Nar*, *Nap*, *NR*, and *Nas* (Figure 4). The total abundance of these genes was about 350 ppm to 700 ppm in the groundwater. Generally speaking, there were more nitrate reductase genes in downstream than upstream groundwater. It is likely that there were higher concentrations of hydrocarbons. Nitrate reduction is always considered to be heterotrophic metabolism, which needs organic carbon as its carbon source and energy [46].

In subsequent steps of nitrite reduction, there are three steps in denitrification and only one step in dissimilatory and assimilatory reduction. In denitrification, nitrite is reduced to nitric oxide through nitrite reductase encoded by the *NirK* or *NirS* gene, then reduced to nitrous oxide through nitric oxide reductase encoded by the *NorBC* gene, and then reduced to nitrogen through nitrous oxide reductase encoded by the *NosZ* gene. The gene abundance at each step was ~300 ppm, ~200 ppm, and ~90 ppm, respectively. NA68 had the highest gene abundance among all samples in these steps. That may be because there was the highest concentration of organics (COD: 482 mg/L), the lowest concentration of DO (1.02 mg/L), and adequate nitrate (33.21 mg/L) in the NA68 well. Denitrifying bacteria are always considered to be anaerobic heterotrophic microorganisms [47], and therefore suitable for growing in less DO and more organic conditions. There are more denitrification genes involved in the transformation of nitrite to reduced nitrogen upstream and downstream of the aquifer rather than the contamination source zone. This may be caused by less nitrate in the contamination source zone.

In the dissimilatory reduction process, nitrite is transformed into ammonium directly through nitrite reductase encoded by *NirBD* and *NrfAH* genes. The abundance of the two genes was about 100 ppm to 300 ppm, which was greater than that of the *NirK* (*NirS*) gene in denitrification except in the NA68 sample. There was more abundance in upstream and downstream samples. There was less of the assimilatory reduction gene, *NirA*, whose abundance was 0–50 ppm.

Based on the above results, it can be speculated that nitrite reduction was mainly caused by dissimilatory reduction and denitrification in the study aquifer. It is reported that denitrifying microorganisms are capable of oxidizing toluene, ethylbenzene, and m-, o-, p-xylenes, and there was substantial investigation into BTEX degradation under denitrification conditions in aquifers [48,49]. Denitrification is also commonly desired for in situ bioremediation of PHC-contaminated aquifers, therefore nitrate was injected into several PHC-contaminated aquifers to stimulate and enhance denitrification [43,50]. However, dissimilatory nitrate reduction was not regarded as an important process in many contaminated aquifers [49,50,51]. This did not agree with the findings in the study. Studies found that most obligately anaerobic nitrate-reducing bacteria perform dissimilatory nitrate reduction to ammonia (DNRA) rather than denitrification to nitrate [52], and the dissimilatory pathway is regulated by oxygen [53]. It is suggested that most of the nitrate-reducing bacteria in the aquifer are obligately anaerobic.

There were fewer nitrification genes (*AmoABCD* and *Hao* genes) in the aquifer for the limited DO in groundwater [54]. These nitrification genes were more abundant upstream near the contaminated source. That may be because there was some ammonium and relatively abundant dissolved oxygen. Ammonium may not be the limiting factor for nitrification in the aquifer, since there was more ammonium but fewer nitrification genes in NA68 and MW3.

*NxrAB* gene, encoding oxidization of nitrite to nitrate, was abundant in all samples. That is because the *NxrAB* gene shares the same enzyme with *Nar*. The enzyme is nitrite oxidoreductase, which can convert either nitrate to nitrite or nitrite to nitrate [55]. In anaerobic conditions, the enzyme may mostly play a reduction role.

There was also the nitrogen fixation gene (*Nif DHK*) in some wells with little abundance, except NA7 and MW6 wells located downstream, with the least dissolved oxygen, to which nitrogenase is sensitive [56].

To sum up, the dominant nitrogen metabolism in the aquifer is nitrate reduction, which is one of the most important ways to biodegrade petroleum contaminants. Denitrification and dissimilatory nitrate reduction may coexist in the aquifer, and dissimilatory reduction may play a dominant role. Dissimilatory nitrate reduction produces ammonium, which is the preferred nitrogen source for most microorganisms [57]. Therefore, this process can biodegrade hydrocarbon and supply the nitrogen source simultaneously.

#### 3.3.4. Sulfur Metabolism

**Figure 5:**
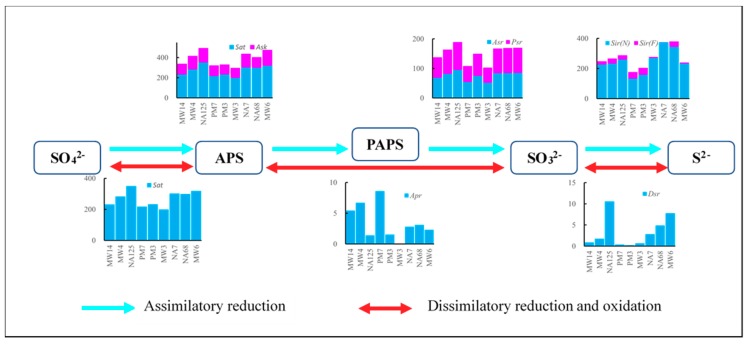
Gene abundance of nitrogen metabolism pathways in the aquifer. APS, adenylyl sulfate; PAPS, 3’-phosphoadenylyl sulfate.

Sulfur metabolism, especially sulfate reduction, plays an important role in hydrocarbon biodegradation [58]. The process of sulfate reduction is always accompanied by biodegradation of hydrocarbons, because sulfate can supply the electron acceptor [21]. The varying abundance of genes relating to sulfate reduction metabolism can be observed between all samples in Figure 5.

There are four steps in assimilatory reduction. The genes encoding the enzymes that transform sulfate to (adenylyl sulfate) APS and then (3’-phosphoadenylyl sulfate) PAPS are *Sat* and *Ask*, whose total abundance was about 300 ppm to 500 ppm. The transformation of PAPS to sulfite genes involves *Asr* and *Psr* genes, which account for about 100 ppm to 200 ppm. The sulfite-to-sulfide transformation genes are *Sir(N)* and *Sir(F)*, which account for 150 ppm to 400 ppm. Interestingly, the abundance of genes involved in each step of assimilatory reduction was less in contamination source area samples (PM7, PM7, and MW3) than other samples. The lower abundance of *Sat* and *Ask* genes in samples from the contamination source area may be caused by the coaction of redox conditions and contaminant concentrations. The *Sat* gene is also involved in sulfur oxidation (Figure 5), which would be an aerobic or nitrate reduction process [59,60]. More *Sat* genes in upstream and downstream wells may be caused by higher DO and nitrate concentrations. Moreover, sulfur oxidation needs reduced sulfur compounds, which would be supplied by the reduction of sulfate in contaminated areas. Therefore, areas with higher DO, nitrate, and contaminant concentrations may have higher *Sat* gene abundance. *Asr* (*Psr*) and *Sir* genes may share the most abundant microorganism, *Novosphingobium*, and other microorganisms with the *Sat* gene (Supplementary Figure S3), which may result in similar distribution among *Sat*, *Asr* (*Psr*), and *Sir* genes.

There are three steps in dissimilatory reduction and oxidation. The *Sat* gene, encoding the first step reaction enzyme, is shared with assimilatory reduction. The second step (APS to sulfite) gene, *Apr*, and third step (sulfite to sulfide) gene, *Dsr*, had negligible abundance (~10 ppm) compared with assimilatory reduction. The abundance of *Apr* and *Dsr* genes was also less in contamination source area samples. Sulfur oxidation microorganisms, such as *Thiomargarita* and *Thiodictyon*, and sulfur-reduction microorganisms, such as *Desulfovibrio* and *Desulfobulbus*, coexist in the aquifer (Supplementary Figure S4). The lower concentrations of DO, nitrate, and contaminants restricted the growth of this kind of microorganism.

Generally speaking, assimilatory reduction genes in the aquifer were more abundant than dissimilatory reduction and oxidation genes. This finding suggests that the biodegradation of petroleum by sulfate reduction is mainly caused by assimilatory reduction in the study aquifer. Assimilatory reduction is the process by which microorganisms take up sulfate to build blocks for peptides and proteins [61]. Therefore, sulfate in the aquifer plays a dual role of electron acceptor and sulfur source.

#### 3.3.5. Other Biodegradation Related Metabolism

**Figure 6:**
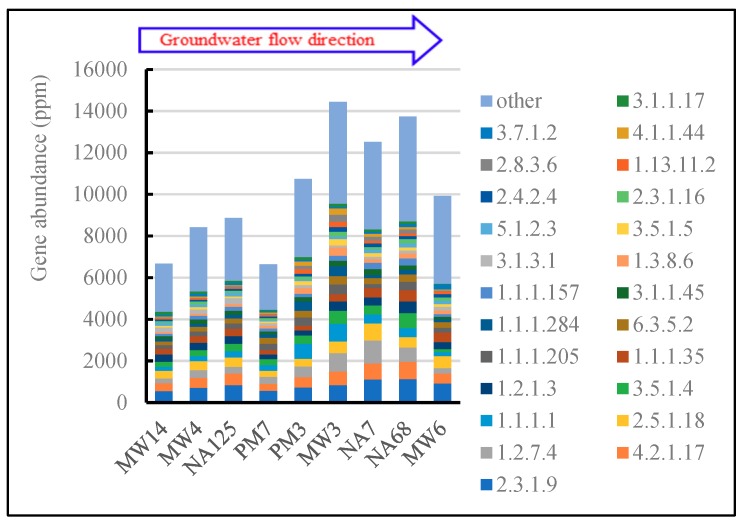
Abundance of genes involved in xenobiotic biodegradation and metabolism.

The components of petroleum contaminants are complex and hard to pick out one by one. Different components may have different metabolic pathways, and there are dozens of enzymes involved in the biodegradation of one component. Moreover, a particular enzyme could be involved in many pathways. Therefore, it is hard to identify the genes involved in the biodegradation of every petroleum contaminant, and all the genes in the xenobiotics biodegradation and metabolism level of the KEGG database were considered as a whole to identify the potential biodegradation ability of petroleum contaminants in the aquifer (Figure 6). There was a higher abundance of genes, greater than 10,000 ppm, in PM3, MW3, NA7, NA68, and MW6 samples, corresponding to the higher concentrations of contaminants (Table 1).

The most dominant genes expressed were acetyl-CoA C-acetyltransferase (EC 2.3.1.9), followed by enoyl-CoA hydratase (4.2.1.17), anaerobic carbon-monoxide dehydrogenase (EC 1.2.7.4), glutathione transferase (EC 2.5.1.18), and alcohol dehydrogenase (EC 1.1.1.1). These are always considered as essential enzymes related to the degradation of benzoate, nitrotoluene, fatty acid, ketone bodies, and some amino acids [62,63,64,65]. The higher abundance of xenobiotic biodegradation genes may indicate higher biodegradation ability in petroleum-contaminated aquifers. In source and downstream areas, there were more contaminants, which may provide the carbon and energy source for the metabolism of biodegradation microorganisms. It was also consistent with the genes related to sulfate and nitrate reduction according to the analysis above.

**Figure 7:**
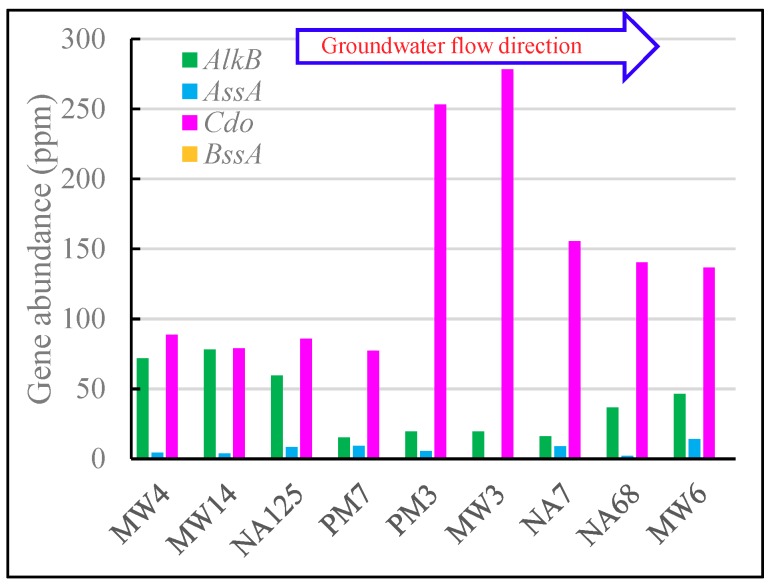
Distribution of *AlkB*, *AssA*, *Cdo*, and *BssA* genes in the aquifer.

The presence of the alkane 1-monooxygenase gene (*AlkB*, EC 1.14.15.3) and catechol dioxygenase genes (*Cdo*, EC 1.13.11.1 and EC1.13.11.2) implies the degradation of medium-chain (C5–C22) alkanes and aromatic hydrocarbons, respectively, using oxygen as an electron acceptor [66,67], while the formate C-acetyltransferase gene (*AssA*, EC 2.3.1.54) and benzylsuccinate synthase gene (*BssA*, EC:4.1.99.11) imply the degradation of chain alkanes and aromatic hydrocarbons, respectively, in anaerobic conditions [13,68]. Figure 7 shows the distribution of these genes in the aquifer.

There were more *AlkB* genes and fewer *Cdo* genes in upstream samples (MW4, MW14, and NA125), and more *Cdo* genes and fewer *AlkB* genes in the contamination source samples (MW3, PM3, and NA7). The downstream samples (NA68 and MW6) had a medium abundance of *AlkB* and *Cdo* genes, while the PM7 sample had the least abundance of both *AlkB* and *Cdo* genes. That may be because there was less contamination and relatively adequate oxygen upstream of the aquifer, and *Cdo* has higher oxygen capture capacity and is more sensitive to the limited oxygen than *Alk B*. Although there was more contaminant in NA7, NA68, and MW6, the amplification of *Cdo* gene was restrained by the limited oxygen.

Although the aquifer was in an almost anaerobic environment, there were fewer anaerobic biodegradation genes. There were no *BssA* genes and fewer *AssA* genes compared with *AlkB* and *Cdo* genes in all samples (Figure 7). The most likely reason is that there is less knowledge about the hydrocarbon anaerobic biodegradation pathway [69]. The genes selected in this study may not be the main anaerobic biodegradation genes. Another probable reason is that anaerobic biodegradation is slower than aerobic biodegradation [70]. There were more *AssA* genes in MW6, NA7, and PM7 for the less dissolved oxygen in these samples.

### 3.4. Possible Mechanism of Biodegradation Related Metabolism

The samples collected in different contaminated areas along the groundwater flow revealed some interesting information about microorganism metabolisms. For examples, in contaminated source zone, there were more abundant genes related with carbon fixation, xenobiotic biodegradation, and catechol dioxygenase, but less alkane 1-monooxygenase gene and sulfate reduction genes.

Based on the information revealed above, possible mechanisms of biodegradation related metabolism could be deduced as follows: Petroleum hydrocarbons stimulate hydrocarbon biodegradation microorganisms, which take nitrate and sulfate as both electrical acceptors and nutrient elements. During biodegradation, some biodegradation microorganisms, considered to be facultative autotrophic bacteria, can use inorganic carbon and intermediate degradation products of contaminant components as their carbon source. The metabolism can fix the inorganic carbon and result in lower DIC concentration in the contaminated source zone. These processes could not generate methane, since the easily metabolized electrical acceptors, nitrate and sulfate, were adequate and there was no methanogenesis condition in the aquifer. This deductive mechanism is based on DNA information, which is a good indication to know the situation in an aquifer. To gain more information on the real activity of genes and the reactions acting in this context, an analysis of mRNA should be carried out in our future study.

## 4. Conclusions

In this study, high-throughput sequencing of environmental DNA was used to gain insight into biodegradation related metabolism in a petroleum-contaminated aquifer. The hydrochemical results suggest that a mass of petroleum hydrocarbons was biodegraded by microorganisms through the metabolism of nitrate and sulfate reduction. Metagenomics analysis proved the speculation and gave more detail information: Nitrate and sulfate reduction were dominated by dissimilatory nitrate and assimilatory sulfate reduction, respectively. This indicates that nitrate and sulfate may serve not only as electron acceptors but also as sources for microorganism metabolism. Although the aquifer was in an anaerobic environment, it had a considerable abundance of aerobic biodegradation genes. Accompanied by biodegradation of hydrocarbons, carbon fixation genes thrived, which probably caused the phenomenon of lower dissolved inorganic carbon concentration in the contaminated source area. Interestingly, no methanogenesis genes were detected in the aquifer, indicating that there was less methane effusing into the atmosphere. Considering the great amount of carbon fixation genes in the aquifer, it can be deduced that there would be a carbon sink effect. These interesting findings motivate us to study the mechanism further.

## Figures and Tables

**Table 1:** Hydrochemical parameters of groundwater samples. COD, chemical oxygen demand; TPH, total petroleum hydrocarbons; VOC, volatile organic compound; BTEX, benzene, toluene, ethylbenzene, m-xylene, p-xylene, and o-xylene; DO, dissolved oxygen; DIC, dissolved inorganic carbon; ORP, oxidation-reduction potential.

Well		MW14	MW4	NA125	PM7	PM3	MW3	NA7	NA68	MW6
Contamination indices	COD (mg·L^−^^1^)	0	0	0	17	27	131	482	295	50
	TPH (μg·L^−^^1^)	640.6	659.8	619.1	6433.7	1872.6	4329	659	13,558	15,280.9
	VOCs (μg·L^−^^1^)	5.7	3	3.5	4.1	2.8	33	1848.7	2807.1	1699.5
	BTEX (μg·L^−^^1^)	2.6	1.7	1.5	1.2	1.4	8	1703.7	1493.5	1461.9
Electron acceptors	DO (mg·L^−^^1^)	4.69	1.89	2.16	1.58	2.42	2.21	1.02	1.79	1.44
	SO_4_^2-^ (mg·L^−^^1^)	237.5	269	123.6	154.9	155.9	107.7	24.68	49.95	51.06
	NO_3_^-^ (mg·L^−^^1^)	116.4	87.6	17.33	2.42	3.54	10.61	33.21	64.97	8.13
Metabolic byproducts	Fe^2+^ (mg·L^−^^1^)	<0.01	0.011	<0.01	<0.01	0.962	1.512	5.164	0.019	3.099
	Mn^2+^ (mg·L^−^^1^)	0.022	0.013	0.271	1.661	2.182	2.145	2.711	0.856	3.139
Other parameters	DIC (mg·L^−^^1^)	248	274	146	187	212	249	292	285	319
	K^+^ (mg·L^−^^1^)	4.41	4.83	2.6	4.32	4.35	4.27	4.06	8.54	3.1
	Na^+^ (mg·L^−^^1^)	115	137.7	125.3	152.1	143.3	155.1	147.4	190.5	123.7
	Ca^2+^ (mg·L^−^^1^)	240	246.4	144.8	165	157.3	122	135.3	87.32	192.9
	Mg^2+^ (mg·L^−^^1^)	83	89	51	67	69	56	62	66	77
	NH_4_^+^ (mg·L^−^^1^)	0	0	0	0	0	27.5	0	300	0
	Cl^–^ (mg·L^−^^1^)	167	202	229	246	202	185	167	475	241
	ORP (mv)	130	203.1	115.8	−35.8	−78.6	−51.9	−98.2	−55.5	−92.7
	pH	6.82	6.75	6.98	6.89	6.90	6.81	6.78	7.20	6.72

**Table 2:** Relative abundance (%) of microorganisms at the domain level.

Domain	MW14	MW4	NA125	PM7	PM3	MW3	NA7	NA68	MW6
Bacteria	98.74	98.56	98.97	97.09	99.20	99.34	98.43	99.50	98.55
Archaea	0.72	0.55	0.24	2.01	0.44	0.07	0.17	0.19	0.10
Eukaryota	0.26	0.49	0.29	0.49	0.25	0.21	0.44	0.17	1.14
Viruses	0.20	0.34	0.45	0.37	0.08	0.36	0.92	0.11	0.20
Unclassified	0.08	0.06	0.05	0.04	0.03	0.03	0.03	0.03	0.02

## Supplementary Materials

The following are available online at https://www.mdpi.com/2076-2607/7/10/412/s1, Figure S1: Gene abundances involved in carbon fixation, nitrate reduction and sulfate reduction, Figure S2: Microorganisms involved in carbon fixation (in Genes level), Figure S3: Microorganisms at genes level in each step of assimilatory reduction and oxidation, Figure S4: Microorganisms at genes level in each step of dissimilatory reduction and oxidation, Table S1: Abundances (ppm) of genes encoding enzymes involved in carbon fixation.

## Abbreviations

AB	Arnon–Buchanan
APS	adenylyl sulfate
BTEX	benzene, toluene, ethylbenzene, m-xylene, p-xylene, and o-xylene
CBB	Calvin–Benson–Bassham
COD	chemical oxygen demand
DH	dicarboxylate–hydroxybutyrate
DIC	dissolved inorganic carbon
DNRA	dissimilatory nitrate reduction to ammonia
DO	dissolved oxygen
EC	electrical conductivity
HH	hydroxypropionate-hydroxybutyrate
HP	3-hydroxypropionate
KEGG	Kyoto Encyclopedia of Genes and Genomes
NCBI	National Center for Biotechnology Information
ORP	oxidation–reduction potential
PAPS	3’-phosphoadenylyl sulfate
PHC	petroleum hydrocarbon contaminated
ppm	Parts per million
VOCs	volatile organic compounds
WL	Wood–Ljungdahl

## References

[1] Verginelli I., Pecoraro R., Baciocchi R. (2018). Using dynamic flux chambers to estimate the natural attenuation rates in the subsurface at petroleum contaminated sites. Sci. Total Environ..

[2] Safdari M.-S., Kariminia H.-R., Rahmati M., Fazlollahi F., Polasko A., Mahendra S., Wilding W.V., Fletcher T.H. (2018). Development of bioreactors for comparative study of natural attenuation, biostimulation, and bioaugmentation of petroleum-hydrocarbon contaminated soil. J. Hazard. Mater..

[3] Ryu J.-H., Dahlgren R.A., Gao S., Tanji K.K. (2004). Characterization of redox processes in shallow groundwater of owens dry lake, california. Environ. Sci. Technol..

[4] Yang J., Li G., Qian Y., Yang Y., Zhang F. (2018). Microbial functional gene patterns related to soil greenhouse gas emissions in oil contaminated areas. Sci. Total Environ..

[5] Alfreider A., Schirmer M., Vogt C. (2012). Diversity and expression of different forms of rubisco genes in polluted groundwater under different redox conditions. Fems. Microbiol. Ecol..

[6] Tischer K., Kleinsteuber S., Schleinitz K.M., Fetzer I., Spott O., Stange F., Lohse U., Franz J., Neumann F., Gerling S. (2013). Microbial communities along biogeochemical gradients in a hydrocarbon-contaminated aquifer. Environ. Microbiol..

[7] Yergeau E., Sanschagrin S., Maynard C., St-Arnaud M., Greer C.W. (2013). Microbial expression profiles in the rhizosphere of willows depend on soil contamination. Isme J..

[8] Main C.E., Ruhl H.A., Jones D.O.B., Yool A., Thornton B., Mayor D.J. (2015). Hydrocarbon contamination affects deep-sea benthic oxygen uptake and microbial community composition. Deep Sea Res. Part I Oceanogr. Res. Pap..

[9] Langille M.G., Zaneveld J., Caporaso J.G., McDonald D., Knights D., Reyes J.A., Clemente J.C., Burkepile D.E., Thurber R.L.V., Knight R. (2013). Predictive functional profiling of microbial communities using 16s rrna marker gene sequences. Nat. Biotechnol..

[10] Stapleton R.D., Sayler G.S., Boggs J.M., Libelo E.L., Stauffer T., MacIntyre W.G. (2000). Changes in subsurface catabolic gene frequencies during natural attenuation of petroleum hydrocarbons. Environ. Sci. Technol..

[11] Stapleton R., Sayler G. (1998). Assessment of the microbiological potential for the natural attenuation of petroleum hydrocarbons in a shallow aquifer system. Microb. Ecol..

[12] Kellermann C., Selesi D., Lee N., Hügler M., Esperschütz J., Hartmann A., Griebler C. (2012). Microbial CO_2_ fixation potential in a tar-oil-contaminated porous aquifer. Fems Microbiol. Ecol..

[13] Beller H.R., Kane S.R., Legler T.C., Alvarez P.J.J. (2002). A real-time polymerase chain reaction method for monitoring anaerobic, hydrocarbon-degrading bacteria based on a catabolic gene. Environ. Sci. Technol..

[14] Chakraborty A., Bhadury P. (2015). Effect of pollution on aquatic microbial diversity. Environmental Microbial Biotechnology.

[15] Pérez-Jiménez J.R., Young L.Y., Kerkhof L.J. (2001). Molecular characterization of sulfate-reducing bacteria in anaerobic hydrocarbon-degrading consortia and pure cultures using the dissimilatory sulfite reductase (dsrab) genes. Fems Microbiol. Ecol..

[16] Reid T., Chaganti S.R., Droppo I.G., Weisener C.G. (2018). Novel insights into freshwater hydrocarbon-rich sediments using metatranscriptomics: Opening the black box. Water Res..

[17] United States Environmental Protection Agency (1996). Method 8260b Volatile Organic Compounds by Gas Chromatography/Mass Spectrometry (gc/ms).

[18] United States Environmental Protection Agency (2003). Non-Halogenated Organics Using gc/fid.

[19] Standard A. (1998). Methods for the Examination of Water and Wastewater.

[20] Zhao Y., Zhang X.-X., Zhao Z., Duan C., Chen H., Wang M., Ren H., Yin Y., Ye L. (2018). Metagenomic analysis revealed the prevalence of antibiotic resistance genes in the gut and living environment of freshwater shrimp. J. Hazard. Mater..

[21] Newell C.J., McLeod R.K., Gonzales J.R. (1996). Bioscreen: Natural Attenuation Decision Support System. User’s Manual Version 1.3.

[22] Ning Z., Guo C., Cai P., Zhang M., Chen Z., He Z. (2018). Geochemical evaluation of biodegradation capacity in a petroleum contaminated aquifer. China Environ. Sci..

[23] American Society for Testing and Materials (2015). Standard guide for remediation of ground water by natural attenuation at petroleum release sites. ASTM. Int..

[24] Suarez M.P., Rifai H.S. (2010). Evaluation of btex remediation by natural attenuation at a coastal facility. Ground Water Monit. Remediat..

[25] Marić N., Matić I., Papić P., Beškoski V.P., Ilić M., Gojgić-Cvijović G., Miletić S., Nikić Z., Vrvić M.M. (2018). Natural attenuation of petroleum hydrocarbons—A study of biodegradation effects in groundwater (vitanovac, serbia). Environ. Monit. Assess..

[26] Bolliger C., Hohener P., Hunkeler D., Haberli K., Zeyer J. (1999). Intrinsic bioremediation of a petroleum hydrocarbon-contaminated aquifer and assessment of mineralization based on stable carbon isotopes. Biodegradation.

[27] Su X.-S., Lue H., Zhang W.-J., Zhang Y.-L., Jiao X. (2011). Super (13) c and super (34) s isotope evidence for biodegradation of a petroleum hydrocarbon-contaminated aquifer in the northeast of china. J. Jilin Univ. (Earth Sci. Ed.).

[28] Ning Z., Cai P., Zhang M., Guo C., Shi C., He Z. (2019). Abnormally Low dissolved inorganic carbon anomaly in petroleum contaminated groundwater caused by microbiological geochemistry. Acta Sci. Circumstantiae.

[29] Slater G.F., Nelson R.K., Kile B.M., Reddy C.M. (2006). Intrinsic bacterial biodegradation of petroleum contamination demonstrated in situ using natural abundance, molecular-level 14c analysis. Org. Geochem..

[30] Alfreider A., Baumer A., Bogensperger T., Posch T., Salcher M.M., Summerer M. (2017). CO_2_ assimilation strategies in stratified lakes: Diversity and distribution patterns of chemolithoautotrophs. Environ. Microbiol..

[31] Hügler M., Sievert S.M. (2011). Beyond the calvin cycle: Autotrophic carbon fixation in the ocean. Annu. Rev. Mar. Sci..

[32] Malkawi H.I., Jahmani M., Hussein E., Al-Horani F., Al-Deeb T. (2009). Investigation on the ability of soil bacterial isolates to degrade petroleum hydrocarbons. Int. J. Integr. Biol..

[33] Sohn J.H., Kwon K.K., Kang J.-H., Jung H.-B., Kim S.-J. (2004). Novosphingobium pentaromativorans sp. Nov., a high-molecular-mass polycyclic aromatic hydrocarbon-degrading bacterium isolated from estuarine sediment. Int. J. Syst. Evol. Microbiol..

[34] Lyu Y., Zheng W., Zheng T., Tian Y. (2014). Biodegradation of polycyclic aromatic hydrocarbons by novosphingobium pentaromativorans us6-1. PLoS ONE.

[35] Kertesz* M., Kawasaki A. (2010). Hydrocarbon-degrading sphingomonads: Sphingomonas, sphingobium, novosphingobium, and sphingopyxis. Handb. Hydrocarb. Lipid Microbiol..

[36] Sheng X., He L., Zhou L., Shen Y. (2009). Characterization of microbacterium sp. F10a and its role in polycyclic aromatic hydrocarbon removal in low-temperature soil. Can. J. Microbiol..

[37] Salam L.B., Obayori O.S., Olatoye N.O. (2014). Biodegradation of anthracene by a novel actinomycete, microbacterium sp. Isolated from tropical hydrocarbon-contaminated soil. World J. Microbiol. Biotechnol..

[38] Ye D., Siddiqi M.A., Maccubbin A.E., Kumar S., Sikka H.C. (1995). Degradation of polynuclear aromatic hydrocarbons by sphingomonas paucimobilis. Environ. Sci. Technol..

[39] Singleton D.R., Ramirez L.G., Aitken M.D. (2009). Characterization of a polycyclic aromatic hydrocarbon degradation gene cluster in a phenanthrene-degrading acidovorax strain. Appl. Environ. Microbiol..

[40] Song W.-F., Wang J.-W., Yan Y.-C., An L.-Y., Zhang F., Wang L., Xu Y., Tian M.-Z., Nie Y., Wu X.-L. (2018). Shifts of the indigenous microbial communities from reservoir production water in crude oil- and asphaltene-degrading microcosms. Int. Biodeterior. Biodegrad..

[41] Li Y., Wu Z., Dong X., Jia Z., Sun Q. (2019). Variance in bacterial communities, potential bacterial carbon sequestration and nitrogen fixation between light and dark conditions under elevated co2 in mine tailings. Sci. Total Environ..

[42] Zhang H., Wang H., Yang K., Sun Y., Tian J., Lv B. (2015). Nitrate removal by a novel autotrophic denitrifier (microbacterium sp.) using fe (ii) as electron donor. Ann. Microbiol..

[43] Cunningham J.A., Rahme H., Hopkins G.D., Lebron C., Reinhard M. (2001). Enhanced in situ bioremediation of btex-contaminated groundwater by combined injection of nitrate and sulfate. Environ. Sci. Technol..

[44] Liu Y., Whitman W.B. (2008). Metabolic, phylogenetic, and ecological diversity of the methanogenic archaea. Ann. N. Y. Acad. Sci..

[45] Jarrell K.F. (1985). Extreme oxygen sensitivity in methanogenic archaebacteria. Bioscience.

[46] Alvarez P., Vogel T. (1995). Degradation of btex and their aerobic metabolites by indigenous microorganisms under nitrate reducing conditions. Water Sci. Technol..

[47] Knowles R. (1982). Denitrification. Microbiol. Rev..

[48] Lovley D. (1997). Potential for anaerobic bioremediation of btex in petroleum-contaminated aquifers. J. Ind. Microbiol. Biotechnol..

[49] Schroth M.H., Istok J.D., Conner G.T., Hyman M.R., Haggerty R., O’Reilly K.T. (1998). Spatial variability in in situ aerobic respiration and denitrification rates in a petroleum-contaminated aquifer. Groundwater.

[50] Schürmann A., Schroth M., Saurer M., Bernasconi S., Zeyer J. (2003). Nitrate-consuming processes in a petroleum-contaminated aquifer quantified using push–pull tests combined with 15n isotope and acetylene-inhibition methods. J. Contam. Hydrol..

[51] Smith R.L., Duff J.H. (1988). Denitrification in a sand and gravel aquifer. Appl. Environ. Microbiol..

[52] Myhr S., Torsvik T. (2000). Denitrovibrio acetiphilus, a novel genus and species of dissimilatory nitrate-reducing bacterium isolated from an oil reservoir model column. Int. J. Syst. Evol. Microbiol..

[53] Tiedje J.M. (1988). Ecology of denitrification and dissimilatory nitrate reduction to ammonium. Biol. Anaerob. Microorg..

[54] Stenstrom M.K., Poduska R.A. (1980). The effect of dissolved oxygen concentration on nitrification. Water Res..

[55] Black E.M., Chimenti M.S., Just C.L. (2019). Metagenomic analysis of nitrogen-cycling genes in upper mississippi river sediment with mussel assemblages. MicrobiologyOpen.

[56] Gallon J. (1981). The oxygen sensitivity of nitrogenase: A problem for biochemists and micro-organisms. Trends Biochem. Sci..

[57] Herrero A., Muro-Pastor A.M., Flores E. (2001). Nitrogen control in cyanobacteria. J. Bacteriol..

[58] Shin W.S., Pardue J.H., Jackson W.A. (2000). Oxygen demand and sulfate reduction in petroleum hydrocarbon contaminated salt marsh soils. Water Res..

[59] Cardoso R.B., Sierra-Alvarez R., Rowlette P., Flores E.R., Gómez J., Field J.A. (2006). Sulfide oxidation under chemolithoautotrophic denitrifying conditions. Biotechnol. Bioeng..

[60] Xia Y., Lü C., Hou N., Xin Y., Liu J., Liu H., Xun L. (2017). Sulfide production and oxidation by heterotrophic bacteria under aerobic conditions. Isme J..

[61] Schiff J., Fankhauser H. (1981). Assimilatory sulfate reduction. Biology of Inorganic Nitrogen and Sulfur.

[62] Ioki M., Baba M., Bidadi H., Suzuki I., Shiraiwa Y., Watanabe M.M., Nakajima N. (2012). Modes of hydrocarbon oil biosynthesis revealed by comparative gene expression analysis for race a and race b strains of botryococcus braunii. Bioresour. Technol..

[63] Ollivier B., Magot M. (2005). Petroleum Microbiology.

[64] Sundberg K., Johansson A.-S., Stenberg G., Widersten M., Seidel A., Mannervik B., Jernström B. (1998). Differences in the catalytic efficiencies of allelic variants of glutathione transferase p1-1 towards carcinogenic diol epoxides of polycyclic aromatic hydrocarbons. Carcinogenesis.

[65] Harayama S., Kishira H., Kasai Y., Shutsubo K. (1999). Petroleum biodegradation in marine environments. J. Mol. Microbiol. Biotechnol..

[66] Vomberg A., Klinner U. (2000). Distribution of alkb genes within n-alkane-degrading bacteria. J. Appl. Microbiol..

[67] Zylstra G.J., Gibson D.T. (1991). Aromatic hydrocarbon degradation: A molecular approach. Genetic Engineering.

[68] Tan B., Dong X., Sensen C.W., Foght J. (2013). Metagenomic analysis of an anaerobic alkane-degrading microbial culture: Potential hydrocarbon-activating pathways and inferred roles of community members. Genome.

[69] Foght J. (2008). Anaerobic biodegradation of aromatic hydrocarbons: Pathways and prospects. J. Mol. Microbiol. Biotechnol..

[70] Kristensen E., Ahmed S.I., Devol A.H. (1995). Aerobic and anaerobic decomposition of organic matter in marine sediment: Which is fastest?. Limnol. Oceanogr..

