# Supplementary material for: Insights into Biodegradation Related Metabolism in an Abnormally Low Dissolved Inorganic Carbon (DIC) Petroleum-Contaminated Aquifer by Metagenomics Analysis
Source: Microorganisms. 2019 Oct 1;7(10):412. doi: 10.3390/microorganisms7100412 (PMC6843334; doi:10.3390/microorganisms7100412)
Supplement: Supplementary file 1 [file microorganisms-07-00412-s001.pdf]

Table S1. Abundances (ppm) of genes encoding enzymes involved in carbon fixation.

Pathway	Enzyme	Description	MW14	MW4	NA125	PM7	PM3	MW3	NA7	NA68	MW6
WL	1.2.7.4	anaerobic carbon-monoxide dehydrogenase	13908	22412	16422	18538	31120	52438	64182	39120	15008
CBB	4.1.1.39	ribulose-bisphosphate carboxylase	924	2138	3026	2754	2124	2958	1200	4112	2734
AB (include DH)	1.1.1.42	isocitrate dehydrogenase (NADP+)	8790	11124	11612	9894	14972	19452	13362	17632	14312
	1.2.7.3	2-oxoglutarate synthase	11932	16012	18158	17922	15722	14176	16546	17032	11016
	1.2.7.1	pyruvate synthase	3518	3648	5452	5132	1610	1578	2908	4424	2708
	4.1.1.31	phosphoenolpyruvate carboxylase	8950	12666	13392	10224	18352	21384	14098	13796	11336
HB/HH	6.4.1.3	propionyl-CoA carboxylase	15072	19836	17128	12134	25370	31004	21360	23798	19622
	6.4.1.2	acetyl-CoA carboxylase	23580	29892	28488	23130	42402	49508	32540	47544	32100

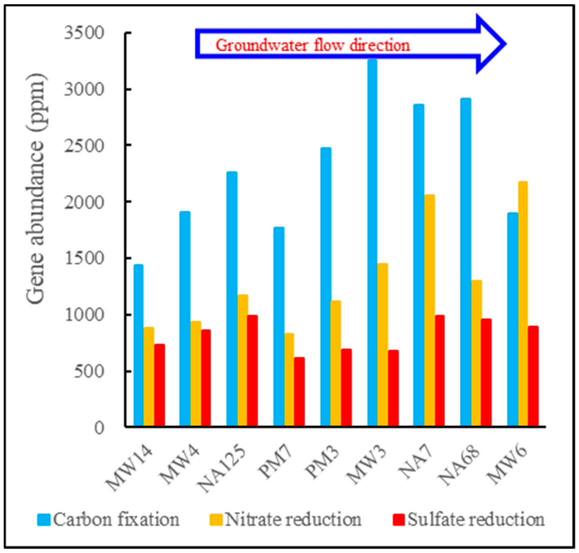

Figure S1. Gene abundances involved in carbon fixation, nitrate reduction and sulfate reduction.

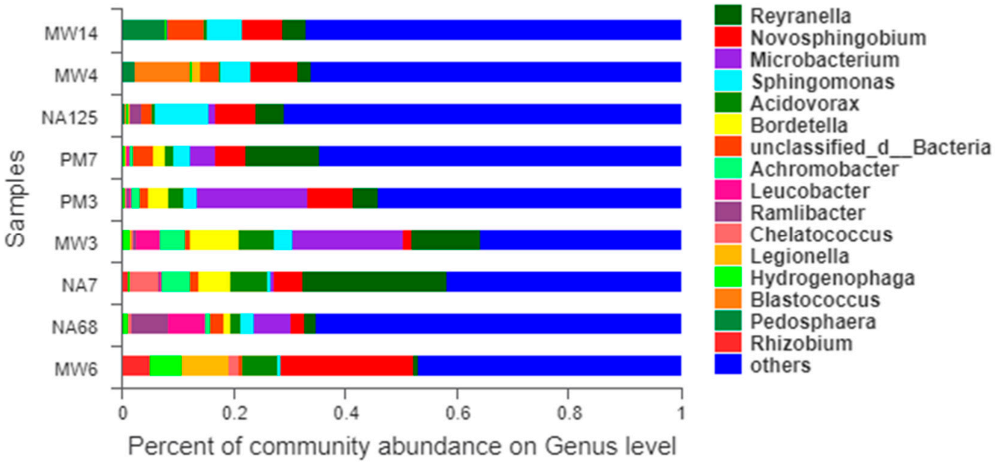

Figure S2. Microorganisms involved in carbon fixation (in Genes level).

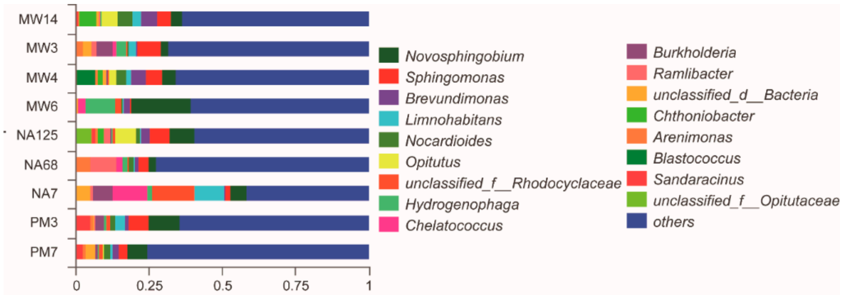

(**a**) *Sat*

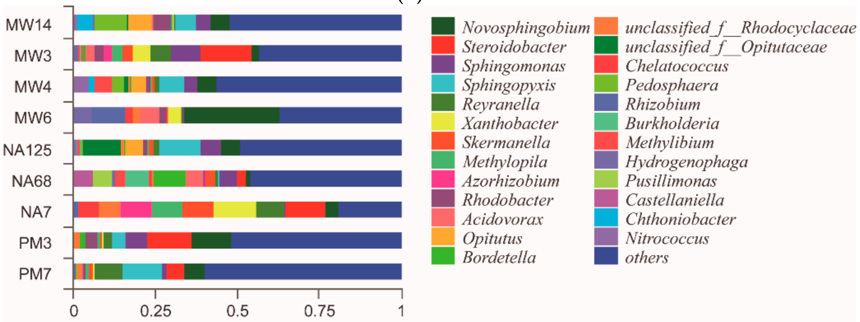

(**b**) *Sir*

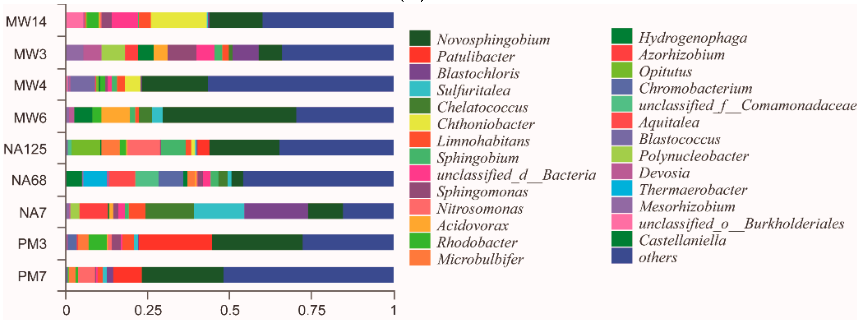

(**c**) Asr (Psr)

Figure S3. Microorganisms at genes level in each step of assimilatory reduction and oxidation.

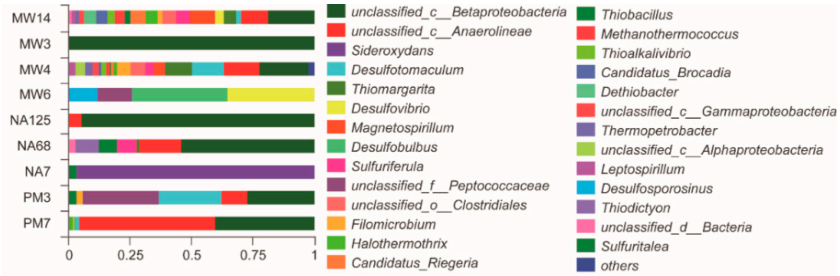

(**a**) Apr

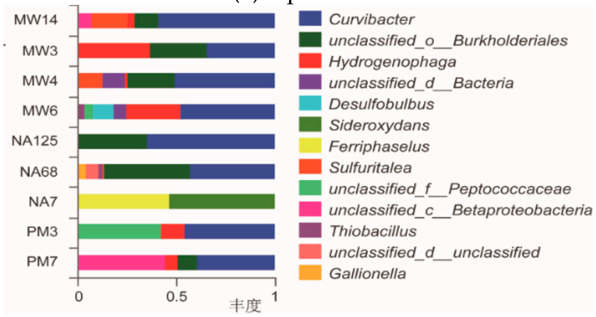

(**b**) Dsr

Figure S4. Microorganisms at genes level in each step of dissimilatory reduction and oxidation.
